# Supplementary material for: A Likelihood Approach for Real-Time Calibration of Stochastic Compartmental Epidemic Models
Source: PLoS Comput Biol. 2017 Jan 17;13(1):e1005257. doi: 10.1371/journal.pcbi.1005257 (PMC5240920; doi:10.1371/journal.pcbi.1005257)
Supplement: S1 File — (TAR.GZ) [file pcbi.1005257.s014.tar.gz › HSPH_Online-SI-Revision/IPoi09/n1-extreme/n1-extreme_BENCH_table-4.pdf]

1	R0	{2.95019, 14.2854}	{2.46519, 15.1347}	{{1.98131, 10.7247}, {2.88523, 18.8445}}	{16.4914, 17.5442}	inf	56.	40.4769	{3., 80.}	59.3132	0.417371	newly	31.	24.5538	{3., 56.}	60.	0.401313	Reff	2.70237	2.32461	{1.79002, 2.74869}	14.9349	ar	924.	868.815	{804., 936.}	6.57842
2	R0	{2.67565, 16.946}	{2.78839, 18.4993}	{{2.56757, 16.6623}, {2.93978, 19.9817}}	{6.42994, 10.873}	inf	194.	200.957	{84., 286.}	31.053	0.160519	newly	79.	81.7714	{42., 108.}	25.7505	0.128608	Reff	1.69101	1.68751	{1.01422, 2.28885}	22.4858	ar	926.	924.629	{898., 944.}	1.50262
3	R0	{2.40066, 16.6543}	{2.34442, 14.8187}	{{1.87927, 9.84456}, {2.81891, 19.8588}}	{12.9811, 19.6768}	inf	86.	83.1563	{22., 150.}	50.327	0.293889	newly	43.	48.0313	{10., 87.}	55.0145	0.279401	Reff	2.02856	1.9619	{1.48468, 2.52622}	16.2317	ar	916.	858.672	{773., 929.}	6.91014
4	R0	{1.9294, 15.4361}	{2.26223, 16.8716}	{{1.88607, 12.6465}, {2.48947, 19.5287}}	{18.4692, 16.8072}	inf	96.	77.9032	{14., 141.}	46.5726	0.321412	newly	51.	37.4355	{7., 70.}	45.0664	0.329038	Reff	1.6207	1.92083	{1.53446, 2.30333}	21.6077	ar	796.	844.532	{778., 898.}	8.1638
5	R0	{2.92859, 19.5393}	{2.04867, 19.4167}	{{1.98971, 19.0665}, {2.11476, 19.6444}}	{30.0459, 0.934286}	inf	172.	119.033	{55., 179.}	33.2348	0.218614	newly	68.	45.6066	{23., 67.}	33.703	0.217208	Reff	1.94751	1.32195	{0.824756, 1.74498}	32.1211	ar	927.	805.787	{761., 841.}	13.0758
6	R0	{1.79622, 12.3148}	{2.03498, 11.5481}	{{1.86868, 9.28761}, {2.28604, 14.5901}}	{13.5251, 15.687}	inf	52.	130.794	{62., 193.}	157.387	0.389325	newly	27.	78.5397	{45., 109.}	193.592	0.450363	Reff	1.38489	1.16656	{0.731568, 1.6462}	27.9929	ar	734.	806.413	{741., 870.}	10.6181
7	R0	{2.45855, 17.1776}	{2.30461, 10.0877}	{{1.7459, 5.83416}, {2.90379, 14.3219}}	{15.5912, 41.2738}	inf	118.	154.547	{56., 260.}	56.9783	0.217462	newly	59.	109.	{54., 153.}	92.4258	0.279151	Reff	1.87833	1.35025	{0.748872, 2.08232}	33.8353	ar	871.	843.625	{697., 931.}	7.73895
8	R0	{2.20009, 15.519}	{2.35288, 18.2777}	{{2.20881, 16.3002}, {2.48947, 19.5287}}	{8.06233, 19.3896}	inf	146.	186.644	{124., 237.}	36.3826	0.147398	newly	70.	68.4068	{50., 88.}	17.9661	0.0918377	Reff	1.44546	0.925838	{0.5188, 1.47784}	39.6408	ar	839.	872.847	{822., 907.}	5.14939
9	R0	{2.06493, 9.6414}	{2.07212, 10.1651}	{{1.31535, 3.47086}, {2.80057, 16.4329}}	{21.8123, 40.4692}	inf	38.	79.7885	{17., 154.}	142.257	0.455465	newly	41.	58.3654	{10., 112.}	78.94	0.386268	Reff	1.89148	1.87502	{1.25218, 2.51239}	19.3513	ar	777.	786.077	{555., 915.}	17.9586
10	R0	{2.60775, 19.6936}	{2.24761, 18.9633}	{{2.20181, 17.1287}, {2.30371, 19.6625}}	{13.8104, 3.73533}	inf	154.	161.438	{94., 214.}	25.832	0.122908	newly	62.	61.3438	{42., 83.}	22.9335	0.112709	Reff	1.84629	1.31502	{0.806869, 1.73539}	30.0343	ar	889.	851.281	{825., 883.}	4.2815
11	R0	{1.99168, 8.13555}	{2.55413, 13.0031}	{{2.15677, 9.98575}, {2.91192, 15.5722}}	{28.2402, 59.8312}	inf	65.	172.552	{42., 274.}	179.61	0.434375	newly	78.	101.985	{26., 149.}	55.3961	0.232813	Reff	1.67899	1.59787	{0.930824, 2.49411}	26.9516	ar	772.	890.567	{833., 936.}	15.3584
12	R0	{1.79304, 9.18941}	{2.32109, 14.3224}	{{1.86868, 9.64595}, {2.78218, 18.4249}}	{31.5508, 59.7391}	inf	28.	34.2097	{2., 89.}	83.5829	0.500381	newly	33.	21.0645	{1., 45.}	60.1173	0.587667	Reff	1.69442	2.25192	{1.81068, 2.6054}	34.2342	ar	725.	812.484	{759., 935.}	26.3003
13	R0	{1.84016, 18.3431}	{1.97767, 16.8018}	{{1.75163, 13.7237}, {2.1968, 19.5406}}	{9.48485, 12.4962}	inf	89.	110.143	{50., 166.}	43.6196	0.176217	newly	37.	47.125	{23., 65.}	42.3263	0.162375	Reff	1.48685	1.39569	{0.983461, 1.69813}	15.6529	ar	762.	784.071	{707., 848.}	7.27878
14	R0	{1.94221, 9.85971}	{2.54164, 14.993}	{{2.02235, 10.6804}, {2.8833, 18.4249}}	{31.3604, 53.7407}	inf	111.	191.157	{79., 285.}	84.157	0.265913	newly	83.	92.9143	{58., 122.}	26.7814	0.119143	Reff	1.44306	1.36483	{0.743645, 2.09105}	29.2258	ar	805.	885.743	{787., 935.}	11.5173
15	R0	{1.78578, 12.0541}	{1.99389, 17.4321}	{{1.77361, 14.2286}, {2.12588, 19.5406}}	{12.2341, 44.6648}	inf	101.	106.536	{33., 171.}	43.2107	0.268818	newly	70.	44.3393	{17., 69.}	37.6276	0.328064	Reff	1.39648	1.42296	{0.931595, 1.86523}	21.4291	ar	732.	774.036	{718., 841.}	10.4118
16	R0	{1.94463, 15.6503}	{2.56087, 13.9327}	{{2.15677, 10.1363}, {2.86118, 16.8013}}	{32.0513, 14.9052}	inf	94.	175.203	{39., 291.}	107.169	0.355505	newly	42.	92.1594	{35., 128.}	131.781	0.381372	Reff	1.60626	1.54904	{0.821159, 2.36332}	27.4966	ar	757.	890.971	{829., 934.}	17.9848
17	R0	{2.08901, 16.4314}	{2.25194, 17.2339}	{{1.96814, 13.609}, {2.45321, 19.5287}}	{10.2514, 12.3814}	inf	110.	86.4603	{9., 156.}	43.7662	0.329818	newly	50.	40.9524	{4., 76.}	40.5079	0.321196	Reff	1.69628	1.84493	{1.43167, 2.30721}	16.6723	ar	826.	833.937	{791., 887.}	6.28003
18	R0	{2.76419, 18.56}	{2.51771, 14.5582}	{{1.95791, 9.82151}, {2.88523, 18.1089}}	{11.096, 21.6377}	inf	102.	159.676	{58., 249.}	76.7882	0.262525	newly	42.	85.9014	{31., 120.}	117.203	0.338282	Reff	2.30257	1.66092	{1.04142, 2.40585}	30.6586	ar	920.	886.479	{790., 936.}	4.58971
19	R0	{2.3821, 17.1399}	{1.626, 5.35126}	{{1.36035, 3.22945}, {2.14082, 9.65117}}	{33.5528, 68.7789}	inf	70.	84.4286	{8., 181.}	72.7211	0.440535	newly	39.	77.619	{13., 121.}	126.862	0.478756	Reff	2.06528	1.03833	{0.686024, 1.48359}	50.5777	ar	883.	634.5	{477., 883.}	28.9516
20	R0	{2.78314, 19.6127}	{2.53918, 19.1551}	{{2.43839, 17.9391}, {2.64373, 19.7855}}	{8.7655, 3.02485}	inf	270.	192.389	{112., 250.}	28.858	0.182111	newly	81.	73.1389	{42., 99.}	20.8162	0.118748	Reff	1.35817	1.31359	{0.695301, 2.03832}	31.6245	ar	943.	895.306	{870., 923.}	5.05774
21	R0	{1.85124, 14.6794}	{1.85166, 12.2373}	{{1.59682, 8.77064}, {2.22731, 17.6755}}	{10.3053, 24.7071}	inf	54.	71.	{24., 134.}	64.1289	0.319943	newly	27.	44.4815	{9., 80.}	88.0658	0.359551	Reff	1.61058	1.48346	{1.0244, 2.02256}	20.1568	ar	730.	711.296	{595., 851.}	13.5058
22	R0	{2.1028, 14.9852}	{2.67684, 15.8068}	{{2.41896, 13.1698}, {2.96978, 18.4249}}	{27.6709, 12.9659}	inf	146.	222.42	{161., 276.}	55.7177	0.189354	newly	80.	96.8841	{77., 118.}	25.5254	0.0989126	Reff	1.46565	1.06793	{0.554675, 1.63317}	33.6038	ar	840.	902.478	{870., 936.}	7.77605
23	R0	{1.86601, 10.2157}	{2.45721, 13.7349}	{{1.88486, 8.39294}, {2.93371, 17.7348}}	{32.7311, 41.1666}	inf	53.	119.942	{31., 218.}	144.135	0.373084	newly	50.	70.9275	{19., 117.}	70.8406	0.27442	Reff	1.64022	1.91821	{1.27022, 2.56449}	27.6094	ar	747.	881.609	{763., 943.}	18.8115
24	R0	{2.34032, 10.0992}	{2.54935, 13.7261}	{{2.03307, 8.88568}, {2.96398, 17.1903}}	{14.6021, 40.2003}	inf	45.	60.8406	{9., 130.}	78.0998	0.320842	newly	41.	42.5942	{7., 86.}	59.1729	0.316385	Reff	2.12969	2.30517	{1.66305, 2.74595}	16.2624	ar	887.	890.275	{813., 940.}	4.5684
25	R0	{1.91548, 15.3401}	{2.31365, 16.8203}	{{2.03251, 13.1693}, {2.52006, 19.5955}}	{21.1748, 17.0533}	inf	62.	111.071	{28., 192.}	102.281	0.360873	newly	31.	51.8429	{10., 90.}	90.553	0.34145	Reff	1.66455	1.76125	{1.10765, 2.19768}	20.5256	ar	765.	848.057	{775., 906.}	13.7591
26	R0	{2.75283, 10.6721}	{2.40247, 15.6513}	{{1.87927, 10.2409}, {2.81891, 19.8999}}	{13.8802, 48.7305}	inf	42.	54.7681	{7., 122.}	82.5052	0.365309	newly	48.	31.2174	{4., 62.}	52.9589	0.423794	Reff	2.52985	2.20412	{1.65383, 2.68376}	14.9019	ar	926.	856.536	{741., 929.}	7.77694
27	R0	{2.08367, 19.7849}	{2.28963, 16.5803}	{{2.03251, 13.0262}, {2.50427, 19.391}}	{11.5896, 16.2116}	inf	122.	167.594	{74., 238.}	54.8924	0.208744	newly	56.	70.2031	{45., 95.}	39.0346	0.157207	Reff	1.4669	1.28537	{0.800046, 1.85193}	26.8871	ar	833.	859.359	{784., 905.}	5.62538
28	R0	{2.64334, 17.1765}	{2.01055, 8.85597}	{{1.69756, 6.01426}, {2.4534, 12.3139}}	{24.4865, 48.5063}	inf	150.	112.175	{51., 198.}	37.3099	0.227155	newly	88.	94.5263	{41., 139.}	30.5024	0.142848	Reff	1.90056	1.36657	{0.734344, 1.97253}	31.5783	ar	934.	791.491	{680., 900.}	15.288
29	R0	{2.87008, 13.3872}	{2.07332, 17.779}	{{1.87975, 14.6061}, {2.26107, 19.5406}}	{27.761, 32.9055}	inf	74.	75.2381	{16., 143.}	51.523	0.326978	newly	61.	33.1905	{6., 60.}	47.515	0.418429	Reff	2.46827	1.74105	{1.40918, 2.02594}	29.4625	ar	941.	800.667	{753., 860.}	14.9132
30	R0	{2.06105, 19.6275}	{2.44388, 19.238}	{{2.41419, 18.743}, {2.50427, 19.5287}}	{18.5747, 1.98464}	inf	138.	168.071	{123., 213.}	26.4493	0.0988903	newly	44.	56.8	{41., 75.}	32.987	0.11864	Reff	1.05732	0.649129	{0.419015, 0.883156}	43.4324	ar	747.	885.043	{861., 911.}	18.4796
31	R0	{2.30764, 13.435}	{1.87532, 7.80089}	{{1.44762, 4.01966}, {2.5684, 13.7324}}	{23.9848, 45.4726}	inf	103.	112.154	{27., 223.}	66.3928	0.384227	newly	60.	78.0769	{34., 128.}	57.0513	0.284058	Reff	1.88303	1.1793	{0.697169, 1.9907}	41.5021	ar	877.	737.385	{517., 911.}	17.9326
32	R0	{2.30565, 11.9237}	{2.05973, 9.66114}	{{1.49267, 4.47101}, {2.7499, 15.6124}}	{19.7613, 33.612}	inf	62.	81.5082	{14., 168.}	80.3279	0.349058	newly	41.	64.8525	{12., 115.}	93.0428	0.344362	Reff	1.97825	1.70029	{0.951086, 2.46725}	23.9345	ar	853.	783.115	{570., 925.}	13.1436
33	R0	{2.24031, 18.582}	{2.02755, 16.9771}	{{1.75163, 13.6186}, {2.26107, 19.5406}}	{9.79463, 12.0665}	inf	83.	79.8644	{15., 149.}	52.0114	0.301795	newly	35.	35.5763	{10., 65.}	48.2324	0.258258	Reff	1.90874	1.63922	{1.23629, 1.92605}	15.3764	ar	864.	784.983	{707., 851.}	9.30242
34	R0	{2.33306, 15.9759}	{2.30721, 13.2411}	{{1.75445, 7.55154}, {2.81891, 18.33}}	{15.4653, 24.386}	inf	138.	95.5246	{23., 186.}	48.2419	0.356663	newly	70.	59.8361	{18., 110.}	45.6206	0.301671	Reff	1.80112	1.87441	{1.12971, 2.57746}	22.792	ar	854.	854.426	{735., 933.}	7.46343
35	R0	{2.48357, 18.3573}	{2.34008, 11.5661}	{{1.83586, 7.22097}, {2.86118, 15.5722}}	{12.8694, 36.9946}	inf	114.	134.891	{47., 241.}	51.028	0.257911	newly	58.	91.75	{35., 140.}	75.2155	0.285943	Reff	1.98686	1.62919	{0.962517, 2.29181}	25.5181	ar	835.	839.719	{743., 927.}	9.11302
36	R0	{2.59548, 19.9046}	{2.25909, 15.5488}	{{1.94282, 12.0157}, {2.53519, 19.3027}}	{13.3418, 21.8857}	inf	110.	104.288	{18., 195.}	44.7304	0.290877	newly	53.	52.9661	{9., 90.}	44.1318	0.275745	Reff	2.02967	1.74338	{1.28142, 2.23401}	17.9848	ar	863.	847.61	{775., 905.}	5.0435
37	R0	{2.29641, 16.9868}	{2.12384, 19.4572}	{{2.11476, 19.2116}, {2.1968, 19.9579}}	{7.51489, 14.672}	inf	149.	138.349	{60., 198.}	26.5367	0.1516	newly	59.	50.8254	{27., 74.}	25.1547	0.142014	Reff	1.61208	1.30583	{0.833345, 1.80797}	24.1589	ar	877.	825.032	{774., 867.}	5.96912
38	R0	{2.65743, 15.4569}	{2.55148, 17.1381}	{{2.18932, 13.2892}, {2.85956, 19.8999}}	{8.61603, 17.3321}	inf	119.	192.352	{104., 262.}	71.7481	0.240903	newly	62.	81.5915	{51., 105.}	43.0486	0.16775	Reff	1.96118	1.39036	{0.793243, 2.01911}	32.5268	ar	897.	891.408	{846., 931.}	3.10424
39	R0	{2.67953, 14.5132}	{2.38948, 15.5556}	{{1.93816, 10.6804}, {2.78218, 19.8588}}	{13.0275, 19.2616}	inf	36.	116.939	{26., 217.}	238.215	0.508499	newly	28.	57.6364	{15., 98.}	124.567	0.375928	Reff	2.47857	1.88097	{1.35492, 2.42884}	24.5228	ar	894.	861.803	{779., 933.}	6.0623
40	R0	{1.94594, 11.928}	{2.54445, 12.5566}	{{2.22671, 10.7276}, {2.91192, 15.278}}	{30.7567, 12.622}	inf	81.	201.6	{138., 272.}	151.434	0.389474	newly	45.	103.692	{76., 138.}	131.795	0.353851	Reff	1.35827	0.937736	{0.5147, 1.68369}	42.2884	ar	796.	896.092	{857., 932.}	12.5744
41	R0	{1.66297, 10.0047}	{1.90168, 14.5069}	{{1.72065, 11.7288}, {2.12978, 17.7287}}	{14.5149, 45.2472}	inf	49.	112.021	{46., 181.}	140.349	0.377946	newly	37.	54.3125	{21., 77.}	65.5968	0.250854	Reff	1.32539	1.19575	{0.774695, 1.69784}	24.218	ar	716.	768.542	{680., 839.}	9.9162
42	R0	{2.91568, 16.4453}	{1.77224, 6.64321}	{{1.37974, 3.3318}, {2.45095, 12.2207}}	{39.217, 59.6042}	inf	83.	71.7955	{14., 180.}	56.3801	0.363369	newly	52.	76.3182	{20., 122.}	72.3776	0.314709	Reff	2.50748	1.48853	{0.912001, 2.20572}	40.7419	ar	919.	685.227	{446., 894.}	25.502
43	R0	{2.55898, 15.8889}	{2.38873, 15.0036}	{{1.87476, 9.82151}, {2.8568, 19.8588}}	{12.5764, 18.4767}	inf	88.	62.1967	{14., 127.}	50.2981	0.36295	newly	64.	34.8033	{8., 61.}	47.6178	0.391533	Reff	2.22119	2.19846	{1.89845, 2.68071}	10.7625	ar	904.	873.705	{805., 933.}	5.35688
44	R0	{2.28649, 15.9017}	{2.63773, 17.051}	{{2.29055, 13.5548}, {2.93978, 19.9817}}	{15.9164, 14.1743}	inf	132.	142.095	{31., 259.}	52.5082	0.283356	newly	71.	66.4865	{19., 112.}	41.3752	0.235712	Reff	1.71487	1.91544	{1.31079, 2.46104}	22.6268	ar	880.	903.216	{857., 940.}	3.98649
45	R0	{2.65121, 18.6339}	{2.48647, 13.622}	{{1.89254, 8.56054}, {2.90165, 17.1903}}	{8.10178, 26.9239}	inf	87.	51.4286	{7., 117.}	55.592	0.506647	newly	50.	36.3016	{5., 83.}	57.746	0.461445	Reff	2.3092	2.28927	{1.81855, 2.84332}	13.5495	ar	928.	864.905	{789., 935.}	7.17193
46	R0	{2.52291, 18.0251}	{2.42608, 17.0993}	{{2.04669, 13.0262}, {2.73517, 19.8588}}	{8.20688, 12.3701}	inf	129.	162.672	{63., 257.}	51.9496	0.212932	newly	55.	73.1343	{32., 103.}	50.7734	0.201934	Reff	1.99814	1.52601	{0.908375, 2.16376}	27.3083	ar	894.	873.299	{807., 923.}	4.19547
47	R0	{2.22618, 17.1225}	{2.38075, 15.5833}	{{1.96373, 11.2959}, {2.78218, 19.8588}}	{12.1365, 16.4885}	inf	75.	74.6769	{16., 145.}	56.0615	0.301239	newly	39.	41.4462	{10., 74.}	55.8185	0.287229	Reff	1.91451	2.06524	{1.67343, 2.54245}	15.7141	ar	850.	865.569	{795., 924.}	5.7267
48	R0	{2.04405, 16.073}	{1.751, 7.17884}	{{1.49267, 4.47101}, {2.31231, 12.2483}}	{19.2449, 55.3957}	inf	64.	77.5882	{22., 147.}	61.3664	0.307004	newly	41.	70.7843	{28., 111.}	90.5308	0.330034	Reff	1.69452	1.1455	{0.711251, 1.75113}	37.151	ar	779.	681.569	{521., 870.}	17.043
49	R0	{2.84396, 18.097}	{1.50193, 3.27025}	{{1.43965, 2.60601}, {1.61691, 4.30394}}	{47.1887, 81.9293}	inf	134.	39.4524	{14., 76.}	70.5579	0.618948	newly	69.	67.881	{21., 133.}	51.9324	0.266944	Reff	2.18132	0.810664	{0.611405, 1.07758}	62.836	ar	952.	581.19	{505., 658.}	38.9506
50	R0	{1.85341, 18.0975}	{1.6476, 4.68426}	{{1.61691, 4.30394}, {1.74592, 5.58477}}	{11.1043, 74.1165}	inf	138.	11.3953	{0., 26.}	91.7425	1.53419	newly	56.	13.6512	{0., 21.}	84.7591	1.13995	Reff	1.27329	0.604143	{0.473415, 0.721142}	52.5527	ar	754.	654.512	{574., 714.}	2.3366
